# Performance and Degradation of Nonwoven Mulches Made of Natural Fibres and PLA Polymer—Open Field Study

**DOI:** 10.3390/polym15224447

**Published:** 2023-11-17

**Authors:** Paula Marasović, Dragana Kopitar, Ružica Brunšek, Ivana Schwarz

**Affiliations:** 1 Department of Textile Design and Management, Faculty of Textile Technology, University of Zagreb, Prilaz baruna Filipovica 28a, 10000 Zagreb, Croatia; dragana.kopitar@ttf.unizg.hr (D.K.); ivana.schwarz@ttf.unizg.hr (I.S.); 2 Department of Materials, Fibres and Textile Testing, Faculty of Textile Technology, University of Zagreb, Prilaz baruna Filipovica 28a, 10000 Zagreb, Croatia; ruzica.brunsek@ttf.unizg.hr

**Keywords:** cellulose fibres, PLA biopolymer, needle-punched nonwoven mulches, degradation, performance

## Abstract

The need for sustainable alternatives to conventional plastic mulches in agriculture has led to the development of various types of biodegradable mulches made from natural fibres and biopolymers to reduce environmental pollution and mitigate soil pollution caused by conventional plastic mulch usage. Degradation, impact on soil temperature and humidity, and weed suppression properties of needle-punched nonwoven mulches of different mass per unit area, made of jute, hemp, viscose, and PLA biopolymer, are investigated. Their biodegradation is determined by changes in the mulch properties (mass per unit area, thickness, air permeability, tensile properties, microscopic images, and FTIR analyses) during 300 days of exposure to the environmental conditions in the period from May 2022 to February 2023. The change in mass per unit area, thickness, air permeability, and tensile properties of nonwoven mulches did not show a tendency to degrade during exposure to environmental conditions. The microscopic and FTIR analysis showed the degradation of the fibres from the mulches during the exposure time to a certain extent. The environmental conditions influence the change in the dimensions of the mulches (shrinkage and expansion)—which impact periodically tested mass results per unit area—as well as their thickness and air permeability. The nonwoven mulches provide higher temperatures compared to bare soil, though not as high as those observed beneath traditional agricultural foil. When comparing the humidity in bare soil and soil covered by mulches during the plant growth period (June to October), it was found that soil humidity was higher beneath all mulches. The nonwoven mulches provide superior soil moisture retention compared to conventionally used agrofoil. Almost all nonwoven mulches effectively suppressed weed growth, except hemp mulches. The newly produced mulches have the potential to replace traditional agrofoil, offering improved conditions for plant growth, effective weed control, and faster degradation without causing harm to the environment.

## 1. Introduction

Mulching is a practice in modern agriculture and horticulture that improves soil fertility, reduces weed growth, conserves moisture, and maintains optimal soil temperature. In agriculture, low-density polyethylene foil mulches (LDPE foil) have been used for more than 50 years. In agriculture, plastic products have increasingly come into use. Annually, for agricultural applications, more than 2 million tons of plastic are used, whereas in Asia and Europe, particularly countries located in the Mediterranean basin, consume the majority of these products. Polyethylene mulch foils, usually after two years of use, must be removed from fields and disposed of, since they lose mulching properties (break down) and do not naturally degrade on the soil [1].

Removal of plastic agricultural mulch foils is time-consuming (about 16 h/ha) and, despite the usage of machines, still requires hand labour [2]. Therefore, the need for sustainable alternatives to conventional plastic mulches in agriculture has led to the development of various types of biodegradable mulches made from natural fibres and biopolymers. 

Biodegradable mulches have the potential to reduce environmental pollution and mitigate soil pollution caused by the use of conventional plastic mulches [3,4]. Conventional plastic mulches have raised environmental concerns due to their non-biodegradable nature and negative impact on soil health. The mulching foil residual, left in the field, may interfere with the plant root development of the subsequent crop. On the contrary, natural mulches maintain the soil’s organic matter, keep the soil loose, and provide food and shelter for earthworms and other desirable soil biota [2]. In recent years, the usage of nonwoven biodegradable mulch has gained popularity in agriculture and horticulture. Biodegradable mulches are usually made from natural and renewable sources, such as jute, flax, and hemp, and are designed to break down in the soil after use. Biodegradability is a crucial aspect of these mulches, since by their degradation soil is enriched and waste is minimised, eliminating conventional mulch’s environmental impact. Biodegradability refers to the capacity of a polymer to break down with the assistance of living organisms into fundamental components, such as water, carbon dioxide, methane, essential elements, biomass, and humic matter [5]. The process of polymer degradation involves the reduction of its molecular weight through chemical reactions such as oxidation, photodegradation, and hydrolysis, facilitated by microorganisms in both aerobic and anaerobic environments. As a result, the polymer experiences a loss of its physical, mechanical, and chemical properties [6]. In response to the negative impact of conventional plastic mulches, researchers are exploring raw materials from natural and renewable sources to produce nonwoven biodegradable mulches as a sustainable alternative.

Research has found that nonwoven biodegradable mulch made from bast fibres, such as flax and hemp, can be an effective alternative to black polyethylene woven mulch for outdoor composting. A study revealed that mulches made of bast fibres can improve soil health, retain nutrients, reduce waste, suppress weeds, and increase plant yield. The degradation of flax and hemp mulches depends on environmental factors such as humidity, temperature, and UV radiation. Higher soil humidity and temperature result in faster mulch degradation. Insects and worms were able to fully degrade the nonwoven fabric made from hemp fibres coated with carbon black-based pigment, making it easy to slice with a garden shovel [7]. Other studies revealed that jute nonwoven mulches increased yield, suppressed weeds, increased moisture and available N, P, and K contents, the organic carbon, available N, P_2_O_5_, and K_2_O to the plant and microbial population of the soil compared to the conventional plastic agrotextiles. Due to providing the necessary microclimate for plant growth by releasing all the mentioned minerals and chemical compounds, jute nonwoven mulch outperformed rice straw and polythene mulches in increasing broccoli yield. Compared with woven fabrics, nonwoven fabrics degrade faster than woven fabrics and lead to faster growth of seedlings as well [8,9,10]. Among cellulose fibres, viscose fibre has become increasingly popular for developing mulches and plant seedling products due to its strong sorption capabilities and rapid biodegradability. Given its porous structure, one of the primary advantages of viscose nonwoven mulch is that it improves soil moisture retention. This is especially useful in dry or desert areas where water conservation is essential for crop development and production. Also, there is evidence that modification of viscose by dyeing, adding a PLA layer to the surface, and incorporating potassium nitrate lead to better biodegradability achieved in terms of weight loss, ultimately eliminating the issue of postharvest mulch disposal [11].

Using mulches, notably nonwoven mulches made from PLA biopolymer, boosts yield and provides higher-quality crops [12,13,14,15]. Research in which PLA mulches were used revealed greater soluble sugars and dry matter levels, and lower concentrations of nitrate ions in tomatoes compared to the un-mulched tomato [12]. Investigation of microbes’ role in PLA degradation obtained in compost and microorganism-rich soil showed that the fastest degradation occurs at elevated temperatures (45 °C and 50 °C). At slightly lower temperatures (25 °C and 37 °C), the degradation rate was low or almost non-existent during 12 months of environmental exposure. The scientists stated that more research is needed to identify the microbes responsible for the accelerated breakdown of PLA [16]. Puchalski et. al. were on a similar track as far as the degradation of PLA biopolymer is concerned. An investigation of the structural changes in PLA spun-bonded mulching nonwovens after two years of outdoor composting showed that degradation of semi-crystalline PLA materials using the outdoor composting method in various climatic conditions is not satisfying. PLA spun-bonded mulching nonwovens degraded slowly and were affected by environmental conditions. The addition of a nitrogen agent slightly accelerated degradation [17]. The PLA nonwoven biodegradation tests performed under laboratory conditions showed that PLA nonwoven fabrics are entirely biodegradable after 16 weeks of biodegradation, with weight loss reaching 100%. The laboratory conditions simulated composting using the mass loss method with constant process parameters, a temperature of 58 ± 2 °C, pH 7, and inoculum humidity W = 52.6%. The nonwoven fabrics subjected to biodegradation had a mass per unit area of about 60 g/m^2^. SEM photos revealed a susceptibility to complete biodegradation under simulated laboratory composting and maximum mass loss [18].

The microporous structure of organic nonwoven mulches allows for light, air, and water to be transmitted to the soil, improving soil structure and enhancing soil nutrient availability, resulting in increased crop yield and quality, unlike plastic film [19]. Aside from raw materials and nonwoven fabric manufacturing technology, mass per unit area and thickness influence soil temperature and humidity fluctuations [20,21]. The critical role in air and water transmission through nonwoven mulch is fabric pore geometries. During the exposure of nonwoven fabrics to external conditions, the fabric is degraded, which changes its structure. Therefore, the positive impact of organic mulches on the soil during a given period is hard to estimate. 

In this study, degradation of needle-punched nonwoven mulches of different mass per unit area, made of jute, hemp, viscose, and PLA biopolymer, and their structural changes during 300 days of exposure to an open field is investigated. Performance, i.e., the nonwoven mulches’ impact on soil temperature and humidity, and their weed suppression properties are analysed. The possibility of conventional agrofoil replacement with investigated biodegradable mulches is determined. 

## 2. Materials and Methods

The field test was realised at Donji Laduc, Croatia (45°53′ N, 15°44′ E), where the climate is humid continental (Dfa) according to Köppen–Geiger’s classification. The experiments were started on 25 April 2022 through the deposition of 7 nonwoven mulch replication plots on the soil, where the last replication plot of nonwoven mulches was collected in February 2023.

The nonwoven mulches were produced on the same technological line, with the same production parameters. The line included a web forming on the card, and web bonding using the needle-punching process (Table 1).

Mulches were produced from hemp, jute, and viscose fibres supplied by Derotex NA and polylactide fibres, PLA fibres, from corn starch (NatureWorks BV, Plymouth, MN, USA) in a nominal mass per unit area of 200 g/m^2^ and 400 g/m^2^. The performance and degradation of newly produced nonwoven mulches were compared with conventional agrofoil that was included in the field experiment. The labelling of samples is shown in Table 2.

**Figure 1:**
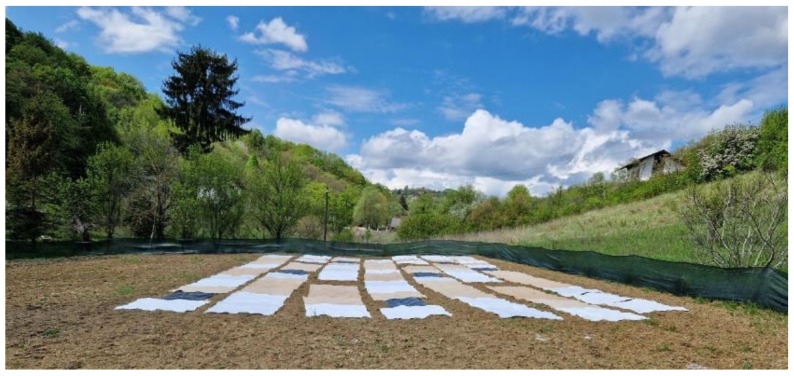
The field experiment with nonwoven fabric mulches.

The mass per unit area of nonwoven mulch made from hemp and jute was 470 g/m^2^, that of regenerated viscose fibres was 200 g/m^2^ and 410 g/m^2^, while the mass per unit area of PLA biopolymer fibres mulch was 250 g/m^2^ and 360 g/m^2^. A commercially available PE agrofoil mass per unit area of 28.17 g/m^2^ and a control field (uncovered field) were included in the field experiment to compare outcomes with traditionally used mulching materials and un-mulched soil. The nonwoven mulches and agrofoil of 2.25 m^2^ (1.5 m × 1.5 m) were placed on the soil in randomly arranged blocks of seven replication plots, including the control fields (Figure 1). 

The first four rows or replication plots of nonwoven mulches and PE agrofoil were removed monthly (four months in a row, from June to September) and after that every two months until the end of the experiment (from November to March) to evaluate the nonwoven mulches’ biodegradation and weed suppression ratio during the field trial. In total, the seven rows of nonwoven mulches were removed from the field in the explained time sequence, and one row of mulches not subjected to weathering (zero samples) was tested on performance and biodegradability. During 300 days of trial, soil temperature and moisture beneath mulches on the field were recorded once per week. Degradation of mulches was recorded throughout the physical–mechanical tests described below. 

### 2.1. Nonwoven Mulches Mass per Unit Area

Mass per unit area is tested according to the current standard for nonwoven textiles, ISO 9073-1:2023 [22]. The five samples were sized to a dimension of 350 mm × 200 mm. Using an analytical balance with a precision of ±0.0001 g, the mass of the samples was measured. The mass per unit area (g/m^2^) was determined according to the obtained mass of nonwoven fabrics, expressed per square meter.

### 2.2. Nonwoven Mulch Thickness

The procedure for “normal nonwoven fabrics” thickness was used, that is, for nonwoven fabrics that are compressible up to 20%. The pressure applied was 1 kPa. A total of ten samples for each nonwoven sample were prepared and tested according to the standard ISO 9073-2:2003 [23].

### 2.3. Nonwoven Mulches’ Air Permeability

The air permeability of nonwoven fabric is measured as the speed of the airflow passing vertically through a nonwoven fabric in specific and set conditions. The nonwoven fabrics were measured on the Air Tronic device, (Mesdan S.p.A, Puegnago del Garda BS, Italy), according to the ISO 9073-15:2008 standard [24]. A flow rate of 10 litres per minute was used, regulating airflow until the desired pressure drops of 100 Pa were achieved, using a circular test area of 10 cm^2^. Results were expressed in cm^3^/cm^2^/s, and statistically processed. Five samples were tested for each type of nonwoven fabric.

### 2.4. Nonwoven Mulch Tensile Properties

The mulches’ breaking force and elongation at the break are determined according to the standard for nonwoven fabric tensile strength and elongation at the break on wide strips (ISO 9073-3:2023) [25]. The five samples per mulch type in the machine direction (MD) and five samples in the cross-machine direction (CD), with dimensions of 350 mm × 200 mm, were tested. The mulches were tested on tensile tester Tenso Lab 5000 (Mesdan S.p.A, Puegnago del Garda BS, Italy). Tests were performed at a constant speed of 100 mm/min with a pretension of 5 N.

### 2.5. Microscopic Images

The fibres taken from nonwoven mulches, unexposed and after exposure to the environmental conditions, were analysed using a microscope equipped with a 5 MP digital camera and an LCD monitor from Bresser, Germany. The analysis was conducted at two magnification levels, i.e., ×400 for jute and hemp fibres, and ×1600 for viscose and PLA fibres.

### 2.6. FTIR Analysis

The FTIR spectra of both control and fibre from the nonwoven mulches exposed to environmental conditions were acquired using a Fourier-Transform Infrared Spectrometer (Perkin Elmer Spectrum One, Waltham, MA, USA). The analyses were conducted at room temperature and under ambient humidity conditions. The solid samples in their original state were positioned on the ATR crystal, ensuring complete coverage and applying pressure. Spectra were recorded within the range of 4000 cm^−1^ and 650 cm^−1^, utilizing a resolution of 4 cm^−1^. Each spectrum was generated by averaging four individual scans.

### 2.7. Soil Temperature and Moisture beneath Nonwoven Mulches

The soil moisture percentage beneath mulches and on the control field (uncovered bare soil) was measured using a PMS-714 soil moisture meter by Lutron Electronic Enterprise Co., Ltd, Taipei, Taiwan, at a soil depth of 15 cm. The soil temperature beneath mulches and on the control field was measured at the same depth with a LabTherm XL (Dostmann electronic GmbH, Wertheim-Reicholzheim, Germany), a bi-metal dial thermometer with a long probe made of waterproof stainless steel. From the nearest hydrometeorological station, the air temperature and humidity during the exposure of mulches to environmental conditions were obtained.

### 2.8. Weed Suppression

**Figure 2:**
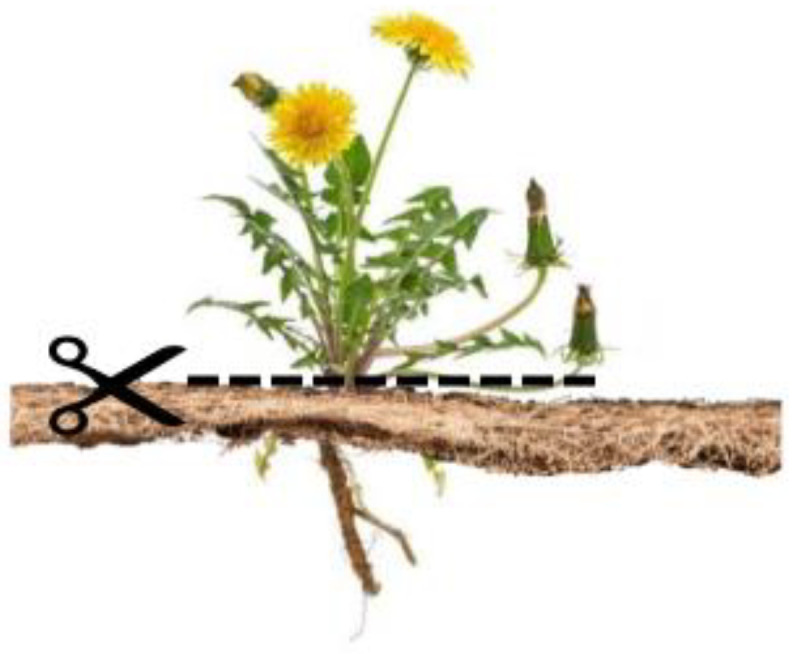
Weed cutting above nonwoven mulches to determine weed suppression ratio.

The ability of the material to suppress weeds was evaluated by comparing the mass of weeds collected on each nonwoven fabric type regarding the mass of weeds grown on the control field, expressed as a percentage. From each nonwoven fabric type, at certain time intervals (at 30, 60, 90, 120, 180, 240, and 300 days), weeds were cut directly above the material (Figure 2), that is, above the soil in the control field.

The collected weeds were dried for the absolutely dry sample and then weighed on an analytical balance. The mass of weeds on the control sample was taken as 100% of weeding, where the percentage of weeding on each nonwoven fabric type and agrofoil was calculated and expressed regarding the weeding of the control field.

## 3. Results and Discussion

### 3.1. Mass per Unit Area, Thickness, and Air Permeability of Nonwoven Mulches

The results of mass per unit area, thickness, and air permeability of nonwoven mulches before exposure to environmental conditions (0 days) and after 30, 60, 90, 120, 180, 240, and 300 days of exposure are presented in Table 3.

The mass per unit area and thickness of nonwoven mulch do not decrease linearly with time, i.e., mass and thickness increase and decrease over time. Although it is assumed that under the influence of weather conditions (temperature, humidity, precipitation) and soil (microorganisms), the decomposition of fibres of the mulches should occur, the degradation of nonwoven fabrics produced on the card and bonded by needle-punching cannot be monitored by mass or thickness reduction. Environmental conditions influence changes in the nonwoven mulches structure, i.e., dimensions, resulting in mulches shrinkage and expansion. Due to nonwoven mulches’ structural changes, air permeability does not show an expected increase due to mulches’ degradation as well. 

**Figure 3:**
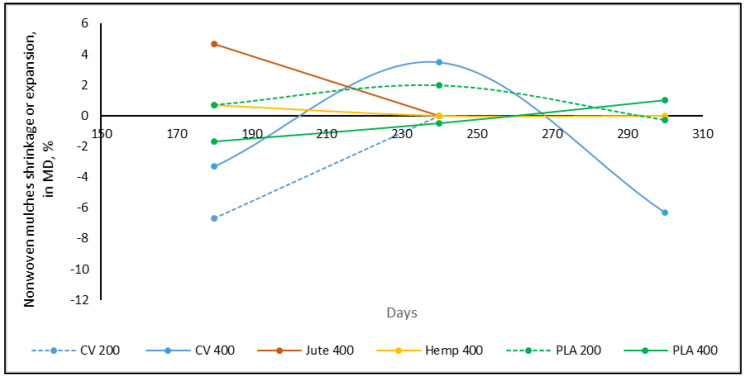
Nonwoven mulches’ dimensional change (%) during exposure period in the MD.

**Figure 4:**
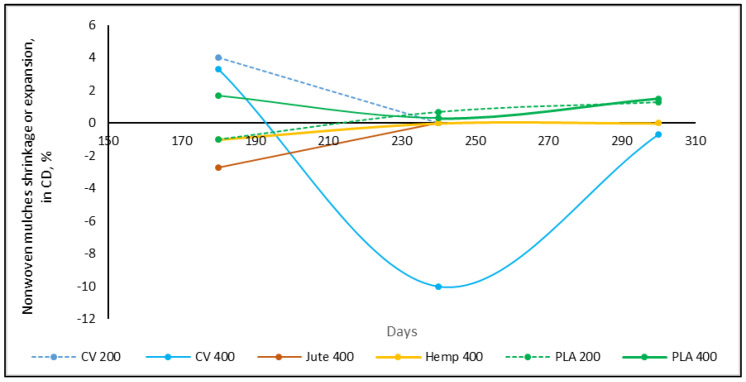
Nonwoven mulches’ dimensional change (%) during exposure period in the CD.

Along with the change in structure, grains of soil, dried weed leaves, and stems are embedded in the mulch structure, between the web layers, increasing the mass and thickness of the nonwoven mulches. As mulches of the same dimensions, 2.25 m^2^ (1.5 m × 1.5 m), were placed on the test field, after removing the mulches from the test site, the dimensions of the mulches were measured, and the percentage of changes in length and width (shrinkage or extension) was calculated (Table 4, Figure 3 and Figure 4).

The trend of the dimension change in the mulches was not established. After 180 days of exposure on the field to environmental conditions, the viscose mulches of 200 gm^−2^ shrank by 6.7% in the machine direction (MD) and extended in the cross-machine direction (CD) by 4.0%, after which the mulch degraded (Table 4). The nonwoven viscose mulches of 400 gm^−2^ after 180 days have the same trend but with a lesser percentage change (mulch in the MD shrank by 3.3% and extended in the CD by 3.3%). After 240 days, mulch extension was 3.5% in the MD and 10.0% in the CD regarding initial dimension (1.5 m × 1.5 m), meaning that after 240 days in total, nonwoven mulches in the MD extended by 6.7% and shrank by 13.3% in the CD. After 300 days, the total change in nonwoven mulch dimensions regarding unexposed mulches to environmental conditions was shrinkage by 6.1% in the MD and 7.4% in the CD.

Mulches made of bast fibres (jute and hemp) have the same trend, where after 180 days nonwoven mulches from bast fibres extended in the MD (4.7%, 0.7%) and shrank in the CD (2.7%, 1.0%). The change in dimension was more pronounced for jute mulches. After 240 days, the jute mulch degraded while the hemp mulch dimension changed to the initial dimension, and finally degraded after 300 days. The dimensional change in PLA mulches produced in different masses per unit area throughout testing differ, where dimensional changes are up to 2.0%.

Dimensional changes in nonwoven mulches should be monitored throughout the exposure period, as some mulches show both dimensional changes, shrinkage and expansion (Figure 3 and Figure 4). An example is viscose mulches of 400 g/m^2^, which after 180 days extended in the CD, then significantly shrank after 240 days, and extended after 300 days (Figure 4). In the case of nonwoven viscose mulches in the MD, the change is reversed, but not so pronounced.

### 3.2. Tensile Properties of Nonwoven Mulches

**Figure 5:**
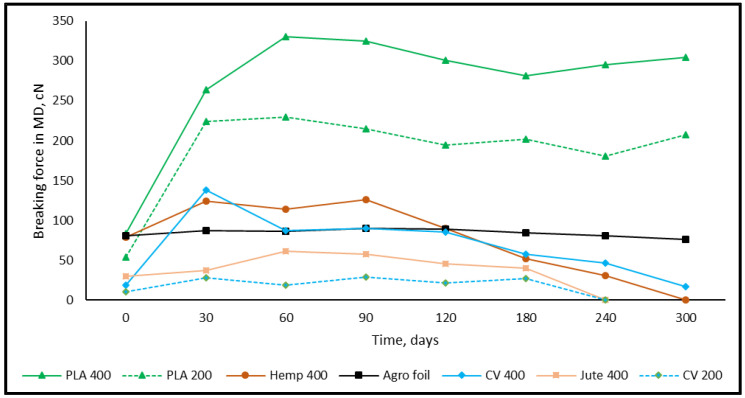
The breaking force of mulches in the MD.

**Figure 6:**
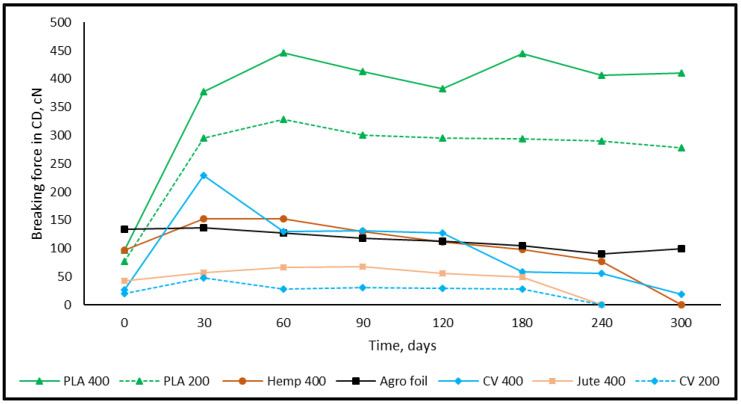
The breaking force of mulches in the CD.

The nonwoven mulches, produced on cards and bonded using the needle-punching process, have a higher breaking force in the CD (cross-machine direction) than MD (machine direction) due to the manufacturing process. The fibre alignment in the cross-machine direction on cross-lappers provides better strength properties in the CD. Considering mass per unit area, the lowest breaking force is in mulches made of viscose followed by jute, hemp, and finally PLA fibres (Figure 5 and Figure 6). 

After 30 days of environmental exposure, a significant increase in the breaking force of all nonwoven mulches in both production directions is visible. The previously mentioned nonwoven mulches’ structural changes influenced the breaking force in the MD and the CD of tested mulches. For example, the mass per unit area and air permeability of mulches made of PLA fibres slightly decrease while thickness slightly increases. Those structural changes provide a higher breaking force of the PLA nonwoven mulch in the MD (315% for mulch of 200 g/m^2^; 217% for mulch of 400 g/m^2^) and CD (284% for mulch of 200 g/m^2^; 290% for mulch of 400 g/m^2^).

The greatest increase in breaking force after 30 days of exposure is visible for viscose mulches of 400 g/m^2^ in both directions (MD for 640%; CD for 775%). Viscose mulches of 200 g/m^2^ have a breaking force increase of 156% in the MD and 148% in the CD. The lowest breaking force increase after 30 days of exposure is visible for bast fibres, i.e., hemp (MD for 57%; CD for 36%) and jute (MD for 23%; CD for 36%). 

Besides mass per unit area and thickness, fibre type has a substantial impact on the nonwoven fabric breaking force. The breaking force of nonwoven fabric is influenced by fibre strength, flexibility, elongation, diameter as well as surface texture, and roughness when a nonwoven fabric is produced using the same technological process and parameters. The impact of fibre arrangement on nonwoven fabric breaking force is pronounced, where it should be noted that different fibre types are differently distributed in the fibrous web. The friction and entanglement between fibres influence the nonwoven fabrics’ breaking force, since some fibre types naturally interlock better, leading to improved breaking force. The reason for the exposed nonwoven mulches’ breaking force increase could be found in the influence of environmental conditions (air and soil temperature and humidity, precipitation) on the structure. Considering the nonwoven mulch fibre types and properties, it can be concluded that the finest viscose fibres (1.78 dtex) change the nonwoven structure after 30 days of exposure the most. Slightly coarser PLA fibres (6.84 dtex) follow viscose mulches, where the coarse bast fibres hemp (58.54 dtex) and jute (31.02 dtex) nonwoven structure changes are the smallest. The breaking force of agrofoil does not follow the trend of the nonwoven mulches’ breaking force.

After 60 days of exposure, the breaking force of viscose mulches decreases in both production directions, and until the end of the testing period (altogether 300 days) slightly decreases and increases. Between 180 and 240 days of exposure, the viscose mulch with a nominal surface mass of 200 g/m^2^ degraded to an extent that it could not be collected from the field and subjected to testing. The decrease in the breaking force of viscose mulch mass of 400 gm^−2^, after 300 days of exposure, is 10.0% in the MD and 27.2% in the CD.

Mulches produced from jute fibres degraded within a period between 180 and 240 days of exposure. Contrary to viscose mulches, the breaking force of jute mulches, in both production directions, just before degradation (tested on mulches after 180 days) was higher than for jute mulches that were not subjected to environmental influences. The above can be explained by the fineness and surface roughness of the jute and viscose fibres. Unlike viscose smooth fibres, the coarser jute fibres retained the nonwoven structure until considerable jute fibre decomposition. 

Hemp mulches were decomposed throughout 240 to 300 days. After 30 days of exposure and a significant breaking force increase, the decrease in hemp mulches’ breaking force tends to decrease in both production directions until their degradation. The breaking force decrease in hemp mulches in the time intervals and during 300 days of environmental exposure is higher in the MD (from 8% to 42%) than in the CD (12% to 21%). The above can be explained by partially decomposed fibres and the frictional forces between the fibres oriented in the CD that maintain the nonwoven fabric structure.

The mulches made of PLA fibres have a lower decrease in breaking force in both production directions (from 1% to 10%). After 300 days of exposure, the breaking force of PLA mulches is significantly higher than before exposure to environmental conditions (from 261.6% to 323.3%) for both production directions. This can be explained by the structural change in nonwoven mulches made of PLA fibres, where the mass per unit area and air permeability did not change significantly and thickness slightly increased.

Average breaking force and elongation at break of the nonwoven mulches tested during the period of exposure to the environmental conditions are presented in Table 5 and Table 6.

Changes in the breaking strength of nonwoven mulches, that indicate structural changes, follow the trends of their breaking forces explained previously. 

After 30 days of environmental exposure, an increase in the breaking elongation of viscose and PLA 200 nonwoven mulches in the MD is visible. In the CD, an increase is visible for all mulches, except CV 200 and Hemp 400. This could be explained by the orientation of the fibres within the nonwoven mulches’ structure, respectively; nonwoven fabrics made on cards have a structure where fibres are mostly orientated in the CD. At 60 days of environmental exposure, change in nonwoven mulches’ elongation at break decreased for almost all mulches, except Jute 400 and PLA 400. After 60 days of environmental exposure, breaking elongation of nonwoven mulches, in both production directions, tends to slightly increase and decrease. At the end of the field experiment, the breaking elongation of all mulches, except mulches made of PLA fibres, significantly decreased in both production directions.

Nonwoven mulches’ tensile properties were tested according to the available standard ISO 9073-3:2023, where five samples were cut and tested in the MD (machine direction) and CD (cross-machine direction). To evaluate the reproducibility of breaking force and elongation at break of nonwoven mulches, the standard error is calculated and provided in Table 6 and Table 7.

### 3.3. Microscopic Images

**Figure 7:**
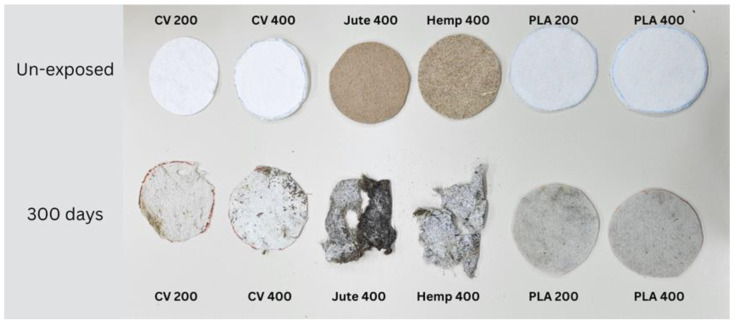
Unexposed nonwoven mulch samples and samples after 300 days of exposure to environmental conditions.

Since the physical–mechanical properties of the nonwoven mulches do not show the expected changes due to decomposition, and the change in structure dimension during exposure is evident, microscopic images of the mulches’ fibres (unexposed and after 90, 180, and 300 days of exposure) were made to investigate the biodegradation of the fibres themselves. The fibres for microscopic images were taken from nonwoven mulches, respectively, from unexposed mulches and after 90, 180, and 300 days of exposure to environmental conditions (Figure 7). 

**Figure 8:**
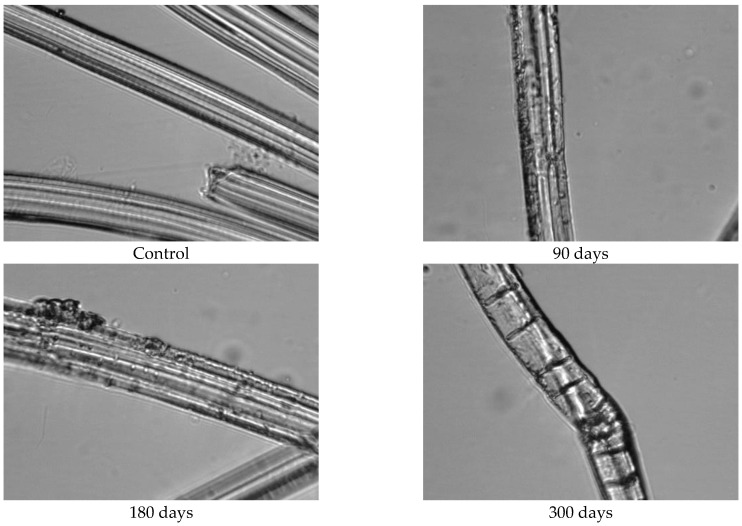
Images of viscose fibres taken from 200 g/m^2^ unexposed nonwoven mulches and those exposed for 90, 180, and 300 days (×1600 magnification).

**Figure 9:**
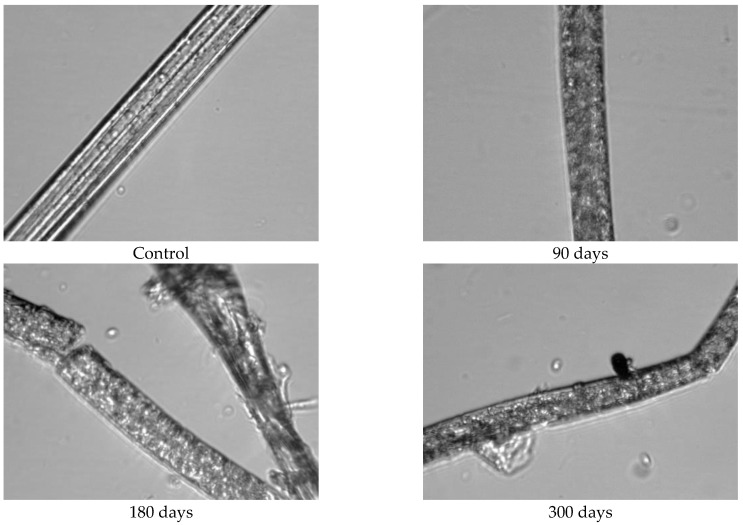
Images of viscose fibres taken from 400 g/m^2^ unexposed nonwoven mulches and those exposed for 90, 180, and 300 days (×1600 magnification).

**Figure 10:**
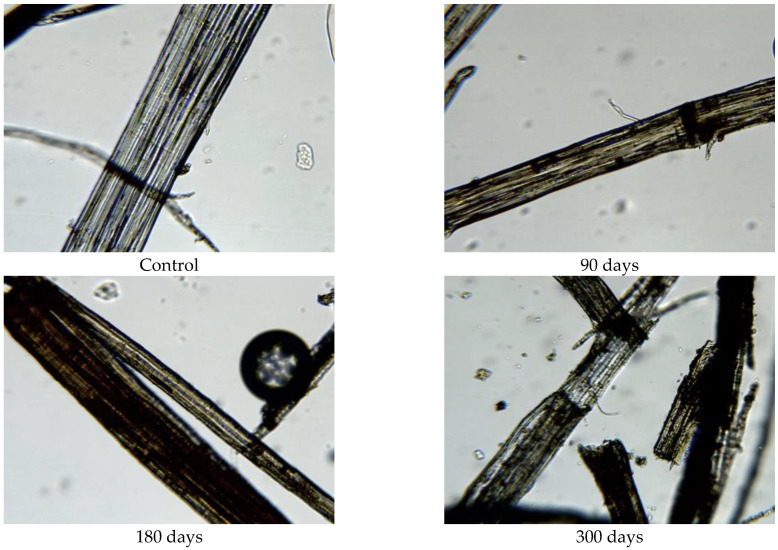
Images of jute fibres taken from 400 g/m^2^ unexposed nonwoven mulches and those exposed for 90, 180, and 300 days (×400 magnification).

**Figure 11:**
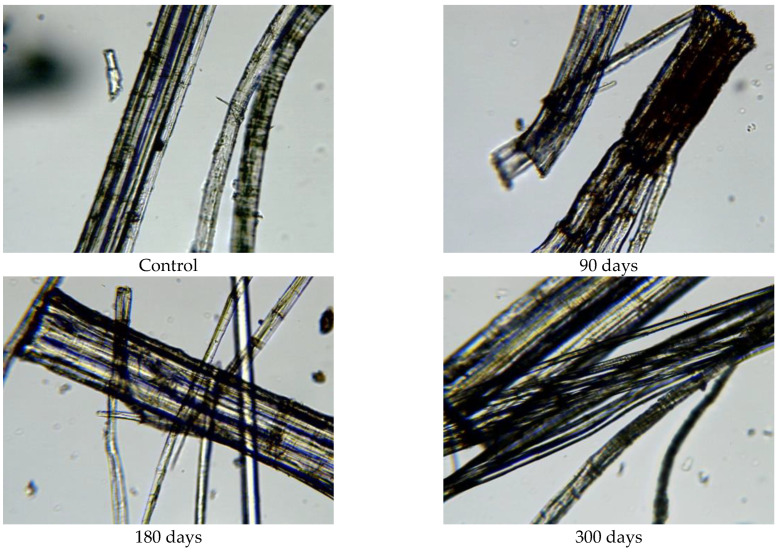
Images of hemp fibres taken from 400 g/m^2^ unexposed nonwoven mulches and those exposed for 90, 180, and 300 days (×400 magnification).

**Figure 12:**
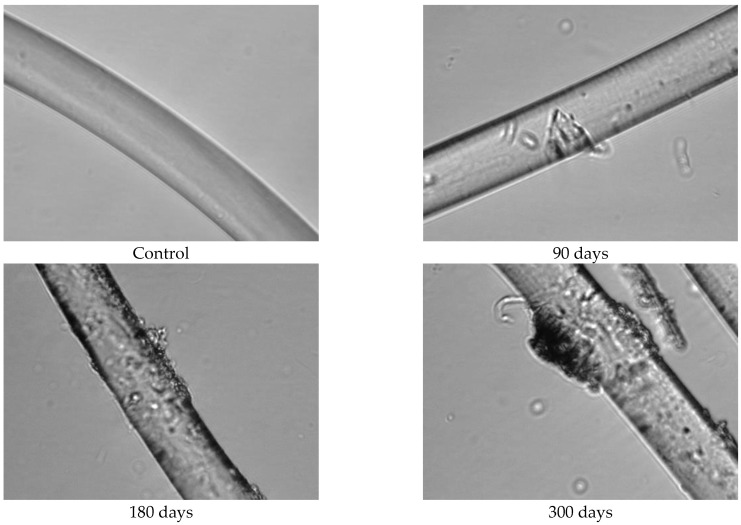
Images of PLA fibres taken from 200 g/m^2^ unexposed nonwoven mulches and those exposed for 90, 180, and 300 days (×1600 magnification).

Morphological analysis and a comparison of the fibre surfaces, as depicted in Figure 7, Figure 8, Figure 9, Figure 10, Figure 11 and Figure 12, reveal significant degradation after 300 days of exposure to environmental conditions. 

Viscose fibre consists of 100% cellulose and exhibits hygroscopic properties, absorbing moisture from the environment. Due to the low degree of polymerisation, weaker orientation, and reduced crystallinity, the fibres tend to characteristically swell and stiffen in the wet state (Figure 7 and Figure 8). Images of unexposed viscose fibres show characteristic longitudinal striations on the fibre surface caused by surface wrinkling during the chemical spinning process, filament hardening, and pulp regeneration. After exposure of viscose mulch of 200 g/m^2^ to environmental conditions, numerous defects become visible on the viscose fibre surface and more pronounced surface cracking and scars are observed after 300 days of exposure (Figure 8). After 90 and 180 days, the protective “skin” created during the spinning process of viscose fibres begins to wrinkle in certain places.

Unexpected results were obtained when comparing viscose fibres from the nonwoven mulches of 200 g/m^2^ and 400 g/m^2^ mass per unit area. Viscose fibres from the 400 g/m^2^ mulches exhibit more significant wrinkling that becomes increasingly pronounced with time exposure to environmental conditions (Figure 9). During the viscose fibre preparation for microscopy, it became evident that there was no need to adhere to the prescribed microscopy procedure, i.e., cutting the fibres. During the nonwoven mulch handling, the fibres from the mulch began to disintegrate, suggesting significant fibre degradation due to exposure to environmental conditions.

The exposed mulch degradation is particularly noticeable for hemp and jute mulches, attributed to the higher moisture absorption capacity of the fibres, which creates favourable conditions for microorganism development. The images of hemp and jute fibres taken from the nonwoven mulches of 400 g/m^2^ exhibit surface damage, roughness, and prominent cracks compared to the control fibres (Figure 10 and Figure 11).

Hemp and jute, as bast fibres, are technical fibres consisting of numerous elementary fibres held together by internal pectin, which is a favoured food source for microorganisms. Since hemp fibres have a lower amount of lignin and pectin, which act as protective layers, compared to jute fibres, it can be assumed that the degradation of hemp fibres started first (Figure 10). The release of elementary fibres from the internal pectin in hemp fibre started after 90 days of exposure to the open field. After 300 days of exposure, hemp fibres predominantly exist as elementary fibres due to microorganism attacks on the internal pectin.

**Figure 13:**
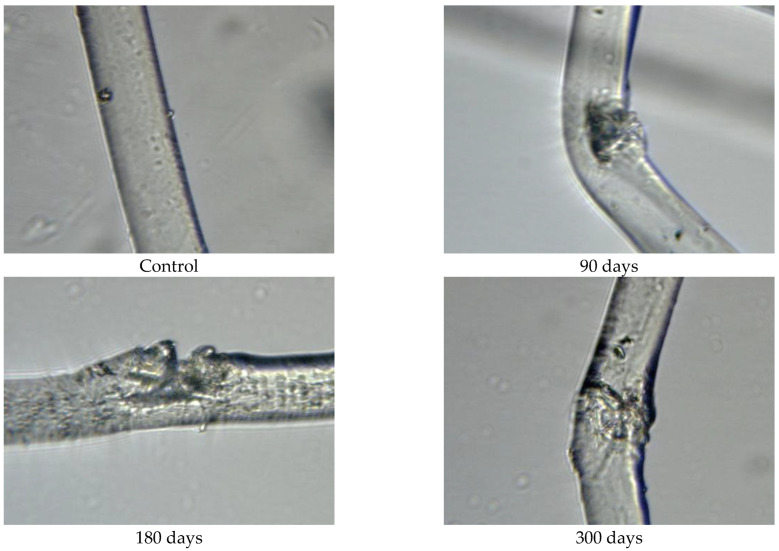
Images of PLA fibres taken from 400 g/m^2^ unexposed nonwoven mulches and those exposed for 90, 180, and 300 days (×1600 magnification).

Before exposure to environmental conditions, the PLA fibres from the nonwoven mulches of 200 g/m^2^ and 400 g/m^2^ (Figure 12 and Figure 13) exhibited smooth and clean surfaces. Comparing the PLA fibres from the 200 g/m^2^ needle-punched nonwoven mulches exposed to environmental conditions for 90 days with the control sample, several cracks were observed on the fibre surfaces, indicating degradation. As the exposure duration extended to 180 and 300 days, the PLA fibres displayed increasing roughness and a higher concentration of cracks on their surfaces as a degradation consequence. Similar behaviour was observed with PLA fibres from the 400 g/m^2^ needle-punched nonwoven mulches, but cracks appeared after 90 days of exposure, increasing with time of exposure to environmental conditions.

### 3.4. FTIR Analyses of Nonwoven Mulches

**Figure 14:**
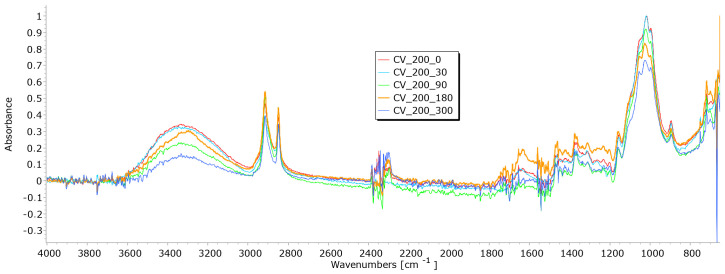
FTIR spectra of CV fibres taken from unexposed nonwoven mulches of 200 gm^−2^ and those exposed for 90, 180, and 300 days.

**Figure 15:**
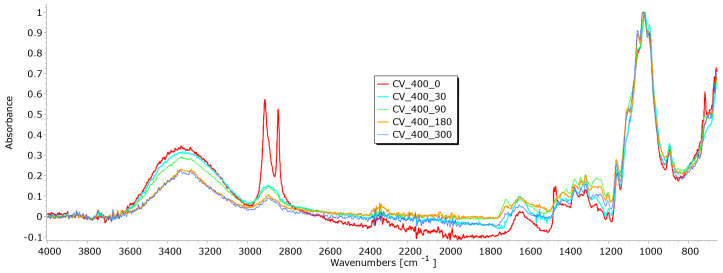
FTIR spectra of CV fibres taken from the unexposed nonwoven mulches of 400 g/m^2^ and exposed for 90, 180, and 300 days.

**Figure 16:**
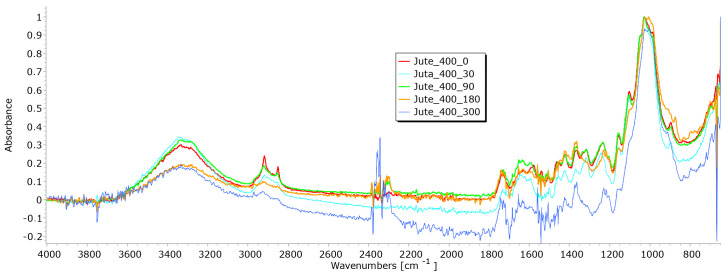
FTIR spectra of jute fibres taken from the unexposed nonwoven mulches of 400 g/m^2^ and those exposed for 90, 180, and 300 days.

**Figure 17:**
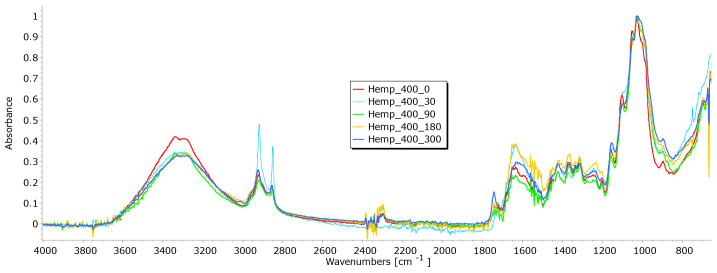
FTIR spectra of hemp fibres taken from the unexposed nonwoven mulches of 400 g/m^2^ and those exposed for 90, 180, and 300 days.

**Figure 18:**
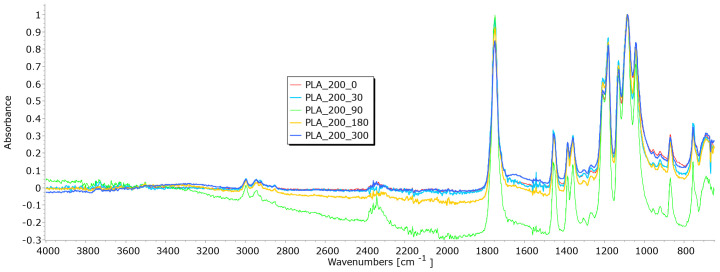
FTIR spectra of PLA fibres taken from the unexposed nonwoven mulches of 200 g/m^2^ and those exposed for 90, 180, and 300 days.

**Figure 19:**
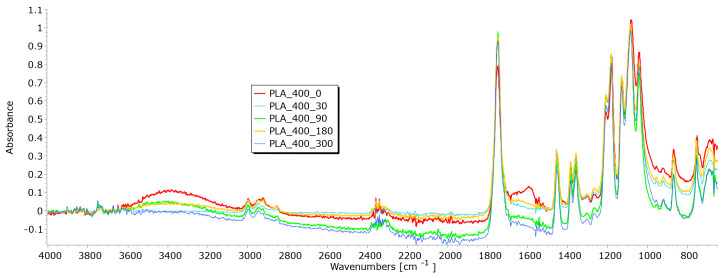
FTIR spectra of PLA fibres taken from the unexposed nonwoven mulches of 400 gm^−2^ and those exposed for 90, 180, and 300 days.

**Figure 20:**
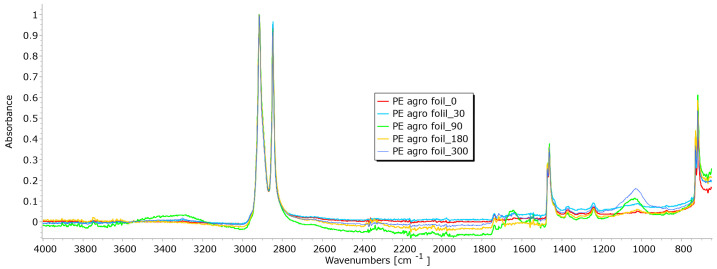
FTIR spectra of unexposed PE agrofoil and PE agrofoil exposed for 90, 180, and 300 days.

From the results of mass per unit area, thickness, permeability, and breaking force, it is notable that the determination of nonwoven mulches’ degradation in time is difficult. Due to the nonwoven mulch structure, the obtained results do not show mulch degradation, just the opposite. To determine to what extent the nonwoven structure impacted the results and degradation of fibre within nonwoven fabric structures occurred, FTIR analysis was performed. The fibres from nonwoven mulches were subjected to Fourier transform infrared spectroscopy, which can provide insights into the changes occurring in the chemical structure of fibres after exposure to environmental conditions (Figure 14, Figure 15, Figure 16, Figure 17, Figure 18, Figure 19 and Figure 20). Comparing the FTIR spectra of the fibres taken from control mulches with nonwoven mulches exposed to environmental conditions, alterations in functional groups and chemical bonds can be identified, indicating progress and extent of degradation. Upon exposure to environmental conditions in an open field, organic material undergoes degradation, resulting in alterations to its chemical composition. Degradation can decrease the intensity of characteristic peaks observed in the FTIR spectra, indicating changes in functional groups or molecular vibrations. After degradation, characteristic peaks of the viscose fibres may increase, while others can remain unchanged, indicating significant alterations in the chemical structure of the fibres. 

Viscose fibres, primarily composed of cellulose, in the 3200–3600 cm^−1^ range exhibit a characteristic peak, representing the stretching vibrations of hydroxyl (OH) groups. Peaks within 2800–3000 cm^−1^ correspond to the stretching vibrations of carbon–hydrogen (C-H) bonds within the aliphatic sidechains of cellulose molecules, while the range of 1000–1200 cm^−1^ reveals stretching vibrations associated with C-O bonds. Additionally, peaks at 1420–1470 cm^−1^ signify the bending vibrations of C-H bonds in the aliphatic sidechains of cellulose. These distinctive peaks not only indicate the presence of cellulose but also provide specific wavenumbers and intensity information that can signal degradation in viscose fibres exposed to environmental conditions [26,27]. 

A decrease in peaks of exposed viscose fibres at higher wavenumbers (3333 cm^−1^, 2915 cm^−1^) signifies modifications or loss of certain functional groups, resulting in changes in molecular vibrations during degradation (Figure 14 and Figure 15). 

Slight changes appear at lower wavenumbers (2916 cm^−1^, 2730 cm^−1^, 2541 cm^−1^), suggesting the formation of new functional groups within the fibre structure. Minor changes observed at lower wavenumbers (under 1000 cm^−1^) may be attributed to signals from other molecules in this region. The region around 3000 cm^−1^, where the hydroxyl band shows changes, is particularly interesting. These changes are linked to alterations in the crystalline structure, indicative of the degradation of the fibres. Analysing viscose fibres from nonwoven mulches of 400 gm^−2^ mass per unit area, a characteristic peak at 2915 cm^−1^ is observed (Figure 15).

With both viscose samples (200 and 400 g/m^2^), it is evident that after 30 days of exposure to external conditions, there was no significant drop in the intensity of the FTIR signals, which were very similar to the zero sample. Increasing the exposure time, this peak changes and decreases slightly, suggesting the progressive degradation of the molecular structure associated with it. Two additional peaks at 2848 cm^−1^ and 2730 cm^−1^ are present in the control sample but disappear in the exposed fibres, signifying significant alterations in the molecular vibrations or functional groups. These FTIR spectral changes provide evidence of the chemical transformations occurring in the viscose fibres during the degradation process (Figure 14 and Figure 15). 

Cellulose is the primary structural component of bast fibres such as hemp and jute, along with a smaller amount of hemicellulose and various other non-cellulosic components. The non-cellulosic components, including lignin, pectin, fats, waxes, water, pigments, minerals, and ashes, are present in varying proportions. In the FTIR spectrum of bast fibres, characteristic peaks associated with cellulosic features appear, such as OH stretching (around 3300–3600 cm^−1^), CH stretching (around 2900 cm^−1^), and CH_2_ bending (around 1200 cm^−1^). In the FTIR spectra of hemp and jute fibres, characteristic peaks typically reflect unique chemical compositions and molecular structures. The exact positions and intensities of the peaks can vary depending on factors such as cultivars, agroecological growing conditions, fibre extraction, mechanical processing, and differences in chemical composition and structure. It is important to note that bast fibres may also contain hemicellulose and lignin, which can introduce additional peaks in the FTIR spectra. The presence and intensity of these peaks can vary depending on the amount of pectin and lignin. It is also necessary to be careful when interpreting the characteristic peaks of lignin and pectin, as they can be closely situated. The characteristic peaks of lignin are typically found in the range of 1600–1700 cm^−1^, representing aromatic ring stretching vibrations, and 830–900 cm−^1^, indicating C-H aromatic bending vibrations. The pectin’s peaks may encompass O-H stretching in the range of 3200–3600 cm^−1^ and C-O stretching in the range of 1000–1200 cm^−1^. To discern between the mentioned compounds, it is essential to identify the specific functional groups associated with each peak. Lignin, for instance, contains aromatic rings, leading to characteristic peaks related to aromatic C=C bonds. Conversely, pectin contains carboxyl groups (C=O), so peaks in the range of 1700–1750 cm^−1^ might indicate the presence of pectin with the fact that lignin’s aromatic ring stretching peaks are usually more intense and prominent compared to pectin’s peaks.

The FTIR spectra of jute and hemp fibres following exposure to environmental conditions reveal noticeable changes in the positions of characteristic peaks and a reduction in peak intensity, indicative of degradation processes (Figure 16 and Figure 17). Changes signify chemical and structural alterations within the fibres, i.e., reduction can be attributed to the breakdown and loss of cellulose chains.

The hemp fibres exhibited a higher reduction in peak intensity, particularly at 1025 cm^−1^ and 1745 cm^−1^, which can be attributed to the higher cellulose content (approximately 70%) and lower lignin content (approximately 6%) compared to jute fibres (55–65% of cellulose and around 12% of lignin) [28,29]. The most significant decrease in peak intensity was observed in the FTIR spectrum of hemp fibres after 300 days of exposure to environmental conditions. The decrease is particularly evident in the peaks at 1025 cm^−1^ and 1745 cm^−1^, which are characteristic peaks of pectin and lignin, which signify a reduction in the concentration of these chemical components. A similar trend was observed in the FTIR spectrum of jute fibres, with the highest reduction in peak intensity occurring after 180 days of exposure to environmental conditions [30,31,32]. 

The FTIR analysis of jute and hemp fibres exposed to environmental conditions reveals significant changes in characteristic peaks, reflecting the degradation and chemical modifications to the fibres. The differing responses of hemp and jute fibres can be attributed to variations in cellulose, lignin, and other components’ content [27,33,34]. 

Several characteristic peaks were identified in the FTIR analysis of the unexposed nonwoven mulch produced from PLA fibres of 200 and 400 g/m^2^ (Figure 18 and Figure 19) [35,36]. The peaks at 3742 cm^−1^ and 3751 cm^−1^ correspond to the stretching vibration of hydroxyl groups (O-H), and the peak at 1750 cm^−1^ (both samples) corresponds to the stretching vibration of the carbonyl group (C=O) in the ester linkage of PLA. In addition, there is a peak at 2997 cm^−1^ and 2998 cm^−1^, corresponding to the stretching vibration of C-H bonds in the methylene (CH_2_) groups of PLA, and peaks at 1180 cm^−1^ and 1179 cm^−1^ corresponding to the stretching vibration of C-O bonds. After fibre exposure of 90, 180, and 300 days to environmental conditions, minimal changes in characteristic peaks were observed, typically considered insignificant and indicative of minor changes in chemical structure. The FTIR spectra of these samples exhibited a slight decrease in peak intensity, suggesting the beginning of fibre degradation.

The main characteristic feature of PE is the intense band associated with the stretching vibration of the methylene (CH_2_) groups. The peak is typically observed around 2916–2848 cm^−1^. The second distinctive peak corresponds to the bending vibrations of CH_2_ groups, which appear around 1470–1465 cm^−1^. The PE polymer also has CH_3_ groups, and their stretching vibrations reveal a peak around 1375 cm^−1^. Peaks associated with the stretching vibrations of carbon–carbon (C-C) and carbon–hydrogen (C-H) bonds in the PE polymer chain can be found in the range of 1100–1200 cm^−1^. These characteristic peaks in the FTIR spectrum of PE agrofoil are the result of its polymer structure, primarily consisting of long chains of carbon and hydrogen atoms. Analysis of the FTIR spectrum of PE agrofoil in this research reveals the absence of significant changes in FTIR peaks (Figure 20). This suggests that even after exposure to environmental conditions for 90, 180, and 300 days, discernible degradation in the PE agrofoil was not obtained. This behaviour is consistent with the known resistance of PE to any environmental conditions, owing to its chemical stability. Additionally, the relatively short exposure time may not have been adequate for the noticeable degradation in agrofoil.

### 3.5. Performance of Nonwoven Mulches—Soil Temperature and Moisture, and Weed Suppression

From May 2022 to February 2023, the soil beneath the conventional agrofoil was higher than on the bare control field from 0.1 °C to 1.4 °C on average (Table 8). Only in February 2023 was the temperature beneath the agrofoil 0.4 °C lower than on the control field. Comparing temperatures beneath the agrofoil and mulches, it is evident that the temperature beneath the nonwoven mulches was mostly lower. An exception was recorded in June for viscose (0.8 °C, 1.7 °C), jute (1.5 °C), and hemp (0.8 °C) mulches; in November for hemp mulches (3.7 °C); and in December for PLA mulches (0.3 °C, 0.4 °C). It can be concluded that nonwoven mulches generally provide higher soil temperatures compared to bare soil, but not as high as beneath conventional agrofoil.

From July to December of 2022, the relative soil humidity was higher beneath the mulches than conventional agrofoil (Table 9). Lower soil relative humidity beneath the mulches regarding agrofoil, considering the period from May 2022 to February 2023, was obtained in May for all nonwoven mulches (from 0.6% to 1.7%), in June for mulches made of PLA fibres (0.6%; 0.5%), in January 2023 for viscose mulches (0.3%) and jute mulches (0.5%), and February for jute (2.3%), hemp (1.0%), and PLA mulches (1.2%). 

In months when plants are growing, higher relative soil humidity provides a better climate for plant development and growth. Considering bare soil humidity and soil humidity beneath the mulches, higher soil humidity was found beneath all mulches in the period of plant growing (June to October) except in May. The produced nonwoven mulches showed better soil humidity maintenance than conventionally used agrofoil.

Sustainable mulches should have similar performance to conventional agrofoil related to weed suppression. To determine the nonwoven mulches’ weed suppression ability, weeds under the mulches and on the control field were collected, dried, and weighed. The percentage of weeds that passed through the mulch relative to weeds grown on the control field was determined (Table 10).

From June to October, weeds only passed through nonwoven mulches produced from bast fibres, where the percentage of weeds that passed through jute mulches was low (up to 2.2%). Relative to the control field, 14.3% of weeds passed through the nonwoven mulches made of hemp fibres. From October, a low percentage of passed weeds were found on viscose and PLA mulches of 400 gm^−2^. In December, the percentage of weeds that passed through the viscose nonwoven mulches increased (10.3%). Considering the obtained results, it can be concluded that the produced nonwoven mulches provided good weed suppression, except the hemp mulches.

## 4. Conclusions

The mass and thickness of nonwoven mulch do not decrease linearly with time, making it challenging to monitor the degradation of nonwoven fabrics produced via needle-punching solely through mass or thickness reduction. Air permeability also does not exhibit the expected increase as mulches degrade. Environmental conditions lead to structural changes in nonwoven mulches, including both shrinkage and expansion.

The breaking force in nonwoven mulches is higher in the cross-machine direction due to fibre orientation during production, with viscose mulch having the lowest breaking force, followed by jute, hemp, and PLA fibres. The trend of breaking force changes during 300 days of exposure remains undetermined. However, the breaking force decreases after an initial increase and eventually reaches degradation. After 240 days, viscose and jute mulches degrade, while hemp mulch degrades after 300 days. PLA mulches exhibit significantly higher breaking forces after 300 days of exposure.

Microscopic and FTIR analyses revealed fibre degradation to varying extents depending on fibre type. Viscose mulches showed enhanced structural integrity and mechanical properties due to crosslinking reactions. Jute and hemp fibres experienced noticeable changes, signifying chemical and structural alterations. Minimal changes were observed in PLA fibres after 300 days.

Changes in mass, thickness, air permeability, and tensile properties of nonwoven mulch did not exhibit a clear degradation trend during exposure to environmental conditions. The layered structure of nonwoven mulches and environmental factors influenced dimensional changes, affecting the mass, thickness, and air permeability results.

Nonwoven mulches maintained higher soil temperatures than bare soil but not as high as conventional agrofoil. Soil humidity beneath the mulches was higher during the growing season (June to October) compared to that of bare soil. Nonwoven mulches generally suppressed weeds effectively, except for hemp mulches.

Newly produced nonwoven mulches have the potential to replace conventional agrofoil, providing better growing conditions, effective weed suppression, and faster degradation without environmental pollution.

## Figures and Tables

**Table 1:** Basic parameters of nonwoven mulch production.

Feeding Speed, m/min	12–22 (Depending on Mulch Mass per Unit Area)
Card speed, m/min	29
Cross-lapper speed, m/min	14.5
Number of web layers	6–16 (depending on mulch mass per unit area)
Pre-needling speed, m/min	4.5
Number of needles in the pre-needling board	7500
Needling speed, m/min	5
Number of needles in the needling board	15,500
Winding speed, m/min	4.6
Working width, m	1.5

**Table 2:** The nonwoven mulch labelling.

Label	Fibre Type	Nominal Mass per Unit Area, g/m^2^
CV 200	Viscose	200
CV 400		400
Jute 400	Jute	400
Hemp 400	Hemp	400
PLA 200	PLA (polylactic acid)	200
PLA 400		400
Agrofoil	Conventional agrofoil	28

**Table 3:** The mass per unit area, thickness, and air permeability of nonwoven mulches before (0 days) and after 30, 60, 90, 120, 180, 240, and 300 days of exposure to environmental conditions.

Mulch	Parameter	Days							
		0	30	60	90	120	180	240	300
CV 200	Mass per unit area, g/m^2^	203.0	132.7	153.6	214.5	195.7	227.0	D	D
	Thickness, mm	3.0057	2.0417	1.7710	2.7261	2.2607	2.5250	D	D
	Air permeability, cm^3^/cm^2^/s	77.5	108.5	86.1	86.6	92.7	82.9	D	D
CV 400	Mass per unit area, g/m^2^	410.8	338.1	320.9	342.4	287.4	308.8	289.2	333.2
	Thickness, mm	6.2356	4.1623	3.5085	3.6715	3.1298	3.3550	3.1417	3.8754
	Air permeability, cm^3^/cm^2^/s	31.5	32.6	41.9	42.3	41.3	35.2	34.2	42.2
Jute 400	Mass per unit area, g/m^2^	468.8	407.5	387.1	387.8	357.9	358.1	D	D
	Thickness, mm	5.1865	6.0426	6.1834	5.5490	5.7218	5.3435	D	D
	Air permeability, cm^3^/cm^2^/s	126.5	162.8	148.3	223.2	175.0	157.8	D	D
Hemp 400	Mass per unit area, g/m^2^	473.4	377.5	374.1	393.0	367.4	411.1	373.8	D
	Thickness, mm	4.1064	4.9731	4.6549	4.8516	5.1634	4.5602	4.9774	D
	Air permeability, cm^3^/cm^2^/s	199.4	257.7	168.9	234.1	230.8	219.8	190.9	D
PLA 200	Mass per unit area, g/m^2^	250.6	232.5	242.3	240.3	211.7	228.2	230.3	240.3
	Thickness, mm	4.0878	4.0912	4.0752	3.9841	4.3793	3.7534	4.4299	4.2813
	Air permeability, cm^3^/cm^2^/s	186.7	184.5	167.7	183.1	198.1	186.6	187.1	173.4
PLA 400	Mass per unit area, g/m^2^	363.9	333.9	351.5	374.8	333.3	363.8	368.1	364.9
	Thickness, mm	5.3825	5.6741	5.7106	6.0134	6.0587	5.7648	5.7697	5.7665
	Air permeability, cm^3^/cm^2^/s	113.3	118.6	121.9	119.4	119.2	116.4	118.5	111.7
Agrofoil	Mass per unit area, g/m^2^	28.2	28.9	29.3	29.4	29.5	26.6	30.4	29.8
	Thickness, mm	0.0670	0.0657	0.0628	0.0589	0.0774	0.1391	0.1259	0.0862

Note: D—degradation

**Table 4:** The nonwoven mulch changes in length (%) and width (%) (shrinkage or extension).

Nonwoven Mulch	Days						In Total	
	180		240		300			
Direction of Production	MD	CD	MD	CD	MD	CD	MD	CD
CV 200	−6.7	+4.0	D	D	D	D	−6.7	+4.0
CV 400	−3.3	+3.3	+3.5	−10.0	−6.3	−0.7	−6.1	−7.4
Jute 400	+4.7	−2.7	D	D	D	D	+4.7	−2.7
Hemp 400	+0.7	−1.0	0.0	0.0	D	D	+0.7	−1.0
PLA 200	+0.7	−1.0	+2.0	+0.7	−0.3	+1.3	+2.4	+1.0
PLA 400	−1.7	+1.7	−0.5	+0.3	+1.0	+1.5	−1.2	+3.5

Note: D denotes degraded mulches; − denotes shrinkage in % to initial dimension; + denotes extension in % to initial dimension.

**Table 5:** Average breaking force and elongation at break of nonwoven mulches in MD and CD before and periodically during exposure to environmental conditions.

	Breaking Force (N) and Elongation at Break (%) in the MD								
Days		0	30	60	90	120	180	240	300
CV 200	N	10.80	27.60	18.50	29.30	21.00	27.12	D	D
	%	17.67	25.54	12.75	13.09	10.00	11.97	D	D
CV 400	N	18.66	138.10	86.70	89.50	85.5	57.96	46.54	16.80
	%	22.27	36.08	29.16	23.26	19.92	4.68	6.91	4.89
Jute 400	N	29.90	36.80	61.20	57.20	45.50	39.78	D	D
	%	21.99	17.91	20.40	21.65	16.74	15.34	D	D
Hemp 400	N	79.10	124.20	114.40	125.62	90.32	52.24	30.65	D
	%	40.85	32.87	28.76	27.50	22.03	10.60	6.10	D
PLA 200	N	53.96	224.10	229.40	215.22	194.46	201.58	180.44	207.70
	%	80.99	86.74	80.06	74.26	75.41	80.76	74.08	78.12
PLA 400	N	83.16	263.64	330.20	324.80	301.00	281.58	295.04	304.94
	%	82.78	81.72	88.55	82.50	79.61	70.71	75.81	75.23
Agrofoil	N	80.22	86.92	85.92	89.76	88.70	84.38	80.76	76.12
	%	197.80	105.62	7.35	5.68	6.00	8.37	5.91	5.91
	**Breaking Force (N) and Elongation at Break (%) in the CD**								
Days		0	30	60	90	120	180	240	300
CV 200	N	19.50	48.40	28.50	30.50	29.60	28.32	D	D
	%	21.67	14.78	8.48	10.69	7.12	7.30	D	D
CV 400	N	26.24	229.50	130.20	131.40	126.90	57.98	56.08	19.10
	%	15.13	22.85	12.20	10.31	8.29	6.94	4.65	4.00
Jute 400	N	42.50	57.6	66.40	68.10	55.60	49.28	D	D
	%	12.44	13.99	13.37	11.92	11.09	11.09	D	D
Hemp 400	N	96.20	151.90	152.40	129.86	111.20	98.00	77.40	D
	%	20.84	19.85	18.57	19.08	13.15	14.89	6.23	D
PLA 200	N	76.76	295.00	328.140	300.60	295.40	293.36	289.42	277.58
	%	65.09	77.49	72.23	65.79	72.16	67.21	64.76	66.54
PLA 400	N	96.88	377.94	446.80	412.82	382.20	444.76	406.34	410.08
	%	55.92	69.45	69.22	68.19	65.95	66.01	66.08	61.74
Agrofoil	N	133.34	135.82	126.92	118.30	112.30	104.74	89.48	99.16
	%	95.33	99.06	81.70	66.04	59.32	49.74	36.15	43.53

**Table 6:** The standard error for breaking force and at break of nonwoven mulches in the MD.

Days	0		30		60		90		120		180		240		300	
MD	F	ε	F	ε	F	ε	F	ε	F	ε	F	ε	F	ε	F	ε
CV 200	1.3	2.2	2.8	1.7	0.7	1.0	2.4	1.5	4.6	2.4	3.2	2.3	D	D	D	D
CV 400	0.7	0.9	7.1	1.4	5.3	2.3	3.4	0.8	3.5	0.9	9.3	0.2	3.6	1.1	9.4	1.1
Jute 400	0.6	0.9	3.5	1.6	4.3	0.9	3.5	1.4	4.4	1.4	5.5	1.5	D	D	D	D
Hemp 400	5.5	1.8	15.7	2.9	17.7	1.4	13.6	1.4	9.1	1.7	9.9	2.2	2.6	0.1	D	D
PLA 200	1.3	3.4	4.4	1.6	14.8	3.0	2.6	2.6	7.3	3.4	5.5	2.0	19.7	5.5	2.9	2.9
PLA 400	3.8	4.2	8.7	2.1	6.4	1.9	6.3	2.0	8.8	1.5	5.6	1.4	14.4	1.9	8.3	3.8
Agrofoil	1.2	116.2	1.1	97.4	1.3	0.3	1.2	0.4	0.6	0.1	0.6	0.7	2.1	0.5	1.6	0.5

Note: F is breaking force; ε is breaking elongation; D is degraded mulch.

**Table 7:** The standard error for breaking force and elongation at break of nonwoven mulches in the CD.

Days	0		30		60		90		120		180		240		300	
CD	F	ε	F	ε	F	ε	F	ε	F	ε	F	ε	F	ε	F	ε
CV 200	0.6	2.1	1.9	0.4	1.9	0.8	2.1	0.6	5.7	1.1	5.4	0.4	D	D	D	D
CV 400	1.6	1.4	6.7	0.6	4.9	0.6	9.1	0.5	8.4	0.4	11.7	0.5	8.6	0.9	2.2	0.1
Jute 400	2.6	0.7	2.9	1.1	4.6	0.4	5.7	0.6	8.4	1.0	6.5	1.1	D	D	D	D
Hemp 400	8.5	1.8	19.4	1.6	20.5	1.7	12.1	1.9	10.2	1.2	11.7	1.1	14.1	1.0	D	D
PLA 200	4.5	1.4	7.5	1.0	5.0	1.5	14.8	1.5	10.6	0.7	17.6	1.9	13.6	3.8	7.0	1.6
PLA 400	3.6	0.8	22.8	2.1	8.2	0.6	34.7	4.9	9.8	0.7	12.5	0.5	11.3	2.7	4.3	1.9
Agrofoil	1.8	7.1	1.8	7.8	1.4	3.9	1.3	4.1	5.5	6.6	2.3	2.2	4.9	6.7	7.0	8.0

Note: F is breaking force; ε is breaking elongation; D is degraded mulch.

**Table 8:** The average monthly temperature (°C) of soil on the control field and beneath mulches in the period of May 2022 to February 2023.

Year	2022								2023	
Month	May	June	July	August	September	October	November	December	January	February
Air T, °C	17.7	22.4	22.9	22.4	15.9	13.0	7.2	3.9	3.7	3.3
CV 200	16.7	22.6	20.6	19.8	16.9	14.2	12.3	5.1	4.5	3.7
CV 400	16.0	23.5	20.2	19.9	16.8	14.0	11.8	4.7	4.2	3.2
Jute 400	17.7	23.3	21.7	20.6	17.2	14.1	12.0	4.9	4.4	3.7
Hemp 400	17.9	22.6	21.5	20.7	17.3	14.3	16.3	5.1	4.5	3.7
PLA 200	18.4	21.3	20.6	20.1	17.2	14.3	12.5	5.5	4.6	3.9
PLA 400	18.3	21.2	20.5	20.3	17.4	14.4	12.7	5.4	4.6	3.8
Agrofoil	18.8	21.8	22.0	20.9	17.6	14.6	12.6	5.1	4.7	4.0
CF, °C	18.6	20.4	20.9	20.1	16.6	13.8	11.8	4.8	4.5	4.4

Note: Air T is the average air temperature in °C; CF is the control field.

**Table 9:** The average relative humidity of soil (%) on the control field and beneath mulches in the period of May 2022 to February 2023.

Year	2022								2023	
Month	May	June	July	August	September	October	November	December	January	February
RH, %	93.0	90.0	90.0	91.0	94.0	94.0	96.0	97.0	97.0	96.0
P, %	53.9	52.0	69.0	22.2	280.0	27.9	119.5	132.3	171.0	27.6
CV 200	24.1	22.6	16.5	15.6	18.7	23.0	23.4	22.1	22.2	20.0
CV 400	24.1	23.5	17.6	16.8	19.3	23.7	24.3	22.7	20.9	19.9
Jute 400	23.8	23.3	17.7	15.3	19.0	23.2	23.6	21.9	20.7	17.3
Hemp 400	23.4	22.6	18.2	15.9	19.4	24.0	23.9	22.5	21.2	18.6
PLA 200	23.0	21.3	15.2	13.7	17.4	22.9	21.0	21.7	21.3	18.4
PLA 400	23.1	21.2	15.5	16.7	18.1	22.6	20.9	21.4	22.4	21.2
Agrofoil	24.7	21.8	12.7	12.1	16.2	20.3	19.9	21.0	21.2	19.6
CF	25.1	20.4	13.8	14.5	17.2	20.6	22.1	23.2	24.6	21.8

Note: RH is average relative humidity of air, %; P is average monthly precipitation in %; CF is the control field.

**Table 10:** Mulches’ weed suppression ability relative to the control field in the period from May 2022 to February 2023.

Mulch	Days					
	June	July	August	October	December	February
CV 200, %	-	-	-	-	D	D
CV 400, %	-	-	-	2.0	10.3	-
Jute 400, %	0.5	1.7	2.2	1.6	D	D
Hemp 400, %	1.6	12.4	14.3	2.0	34.3	D
PLA 200, %	-	-	-	-	-	-
PLA 400, %	-	-	-	1.0	1.0	-
Agrofoil, %	-	-	-	0.2	0.2	-
Control field, %	100	100	100	100	100	100

Note: D—degraded mulches.

## Data Availability

Data are contained within the article.
